# *α*-Pyrone Derivatives from a *Streptomyces* Strain Resensitize Tamoxifen Resistance in Breast Cancer Cells

**DOI:** 10.1007/s13659-017-0136-8

**Published:** 2017-06-20

**Authors:** Rui-Min Yang, Xiu-Lei Zhang, Li Wang, Jian-Ping Huang, Jing Yang, Yi-Jun Yan, Jian-Ying Luo, Xiang-Ting Wang, Sheng-Xiong Huang

**Affiliations:** 1 State Key Laboratory of Phytochemistry and Plant Resources in West China, Kunming Institute of Botany, Chinese Academy of Sciences, Kunming, 650201 People’s Republic of China; 2 Department of Cell and Developmental Biology, School of Life Sciences, University of Science and Technology of China, Hefei, 230026 People’s Republic of China; 3 CAS Center for Excellence in Molecular Cell Science, Chinese Academy of Sciences, Hefei, 230026 People’s Republic of China; 4 University of the Chinese Academy of Sciences, Beijing, 100049 People’s Republic of China

**Keywords:** Breast cancer, Tamoxifen resistance, Resensitization, α-Pyrone derivatives, *Streptomyces*

## Abstract

**Abstract:**

Tamoxifen resistance (TamR) is the underlying cause of treatment failure in many breast cancer patients receiving tamoxifen. In order to look for noncytotoxic natural products with the ability to reverse TamR, an extract from strain *Streptomyces* sp. KIB-H0495 was detected to be active. Subsequent large scale fermentation and isolation led to the isolation of four *α*-pyrone derivatives including two new compounds, violapyrones J (**2**) and K (**3**), and two known analogues, violapyrones B (**1**) and I (**4**). Further bioactivity assays indicated that only **1** and **3** exerted potent resensitization effects on MCF-7/TamR cells at a concentration of 1 μM. Owing to the simple structures of **1** and **3**, these two compounds might have potential for further investigation as novel tamoxifen resensitization agent in breast cancer chemotherapy.

**Graphical Abstract:**

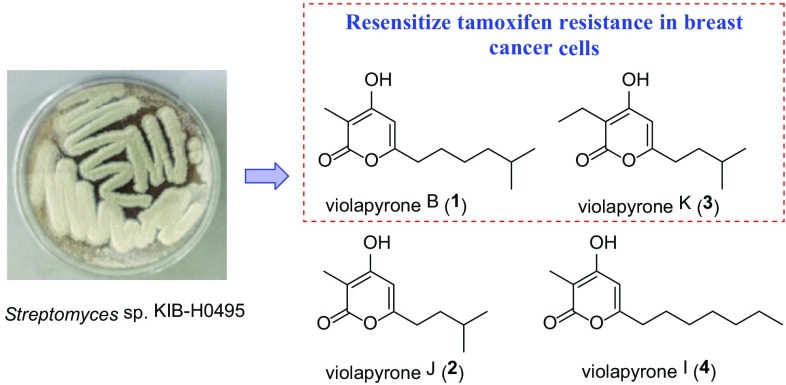

**Electronic Supplementary Material:**

The online version of this article (doi:10.1007/s13659-017-0136-8) contains supplementary material, which is available to authorized users.

## Introduction

Resistance to chemotherapeutic drugs is a severe clinical impediment to the successful treatment in cancer chemotherapy [1]. For the past 30 years, tamoxifen has been the standard treatment for ERα-positive breast cancer in women. However, 30% of patients develop tamoxifen resistance (TamR) [2]. Previous studies suggested that natural products such as vinblastine and puromycin can effectively reverse the drug resistance [3, 4]. It had proved that some small compounds could enhance the cytotoxic effect of tamoxifen on MCF-7/TamR cells, however, a cytotoxicity by itself to MCF-7/TamR cell lines accompanied [5]. Therefore, seeking small molecules with no cytotoxicity is an effective way for resensitizing tamoxifen resistance.

Endophytic strains isolated from the medicinal plants or their rhizosphere are prolific sources of novel natural products [6–9]. It has been shown that these natural products are useful resource to produce pharmacological bioactive agents [10–12]. Nearly 40% of known bioactive compounds with microbial origin are derived from actinomycetes as reported [13–15]. Accordingly, we initiated a program designed to discover bioactive natural products from endophytic actinomycetes in plants from un- and under-explored ecological niches [16–18].

**Fig. 1:**
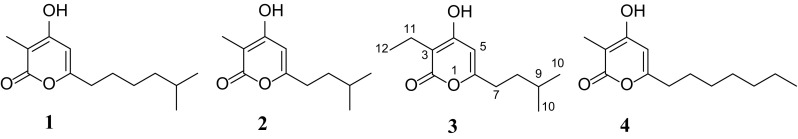
Chemical structures of compounds **1**–**4**

In the present study, we screened extracts library obtained from different actinomycete strains in our laboratory. The results highlighted a crude extract of strain *Streptomyces* sp. KIB-H0495 significantly inhibited resistant strains MCF-7/TamR. Further fermentation and isolation towards this strain led to the identification of four *α*-pyrone derivatives including two new ones, violapyrones J (**2**) and K (**3**), and two known analogues, violapyrones B (**1**) [19] and I (**4**) [20] (Fig. 1). Among them, both violapyrones B (**1**) and K (**3**) exhibited resensitization effect on MCF-7/TamR cells at a low concentration of 1.0 μM. Herein, we described the isolation, structural elucidation and biological activity of violapyrones from *S.* sp. KIB-H0495.

## Results and Discussion

**Fig. 2:**
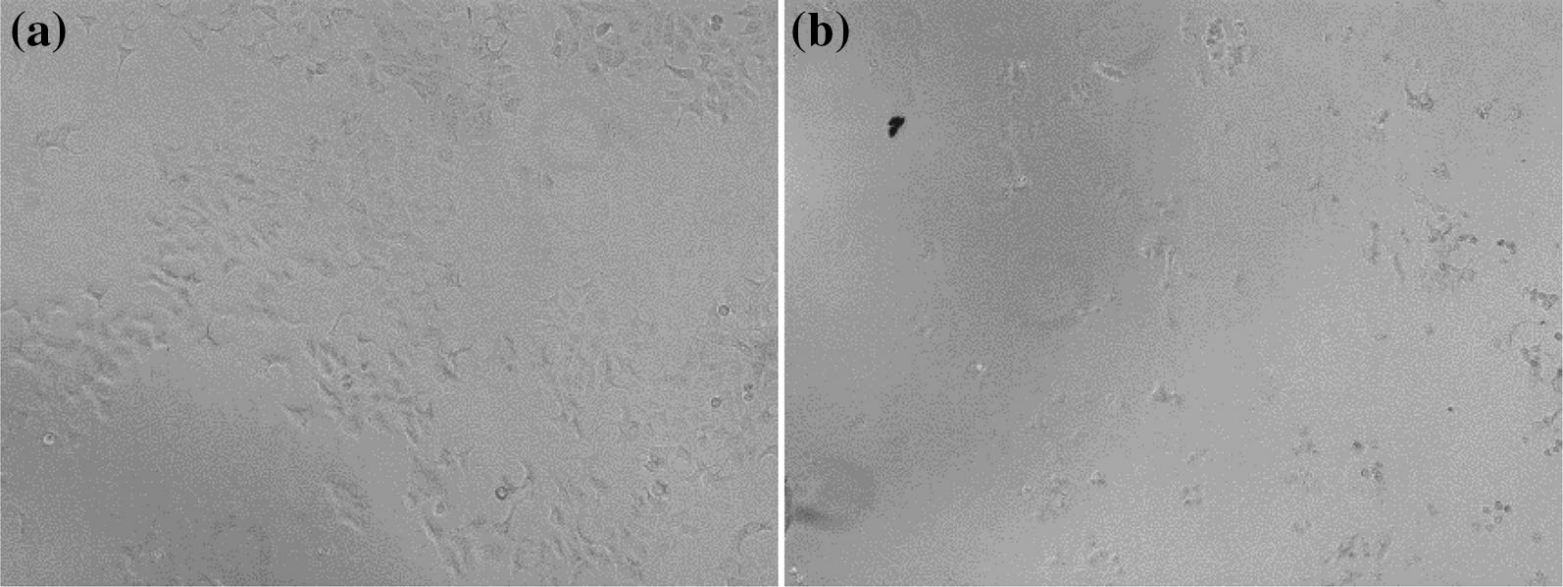
Microscopic observation of MCF-7/TamR treated with DMSO (**a**) and KIB-H0495 extract (**b**) (10 mg/mL, 72 h)

Actinomycete strain KIB-H0495 was isolated from the rhizosphere of plant *Radix stellariae* and was identified as *Streptomyces* based on its morphology and 16S rRNA gene sequence (GenBank no. MF164040) which showed 99.9% identity relative to strain *Streptomyces* sp. G1 (GenBank no. KT252874.1). The crude extract of this strain (10 mg/mL, 72 h) reduced the MCF-7/TamR cell growth by 98.14% (Fig. 2).

Next, the fermentation broth (20 L) of the strain *Streptomyces* sp. KIB-H0495 was centrifuged to obtain supernatant and a mycelial cake, which were extracted with ethyl acetate and acetone, respectively. Both extracts were combined and then applied on repeated silica gel chromatograph column (CC), MCI gel CHP20P column, and semipreparative HPLC to yield four *α*-pyrone derivatives violapyrones B (**1**) [19], J (**2**), K (**3**), and I (**4**) [20] (Fig. 1).

**Fig. 3:**
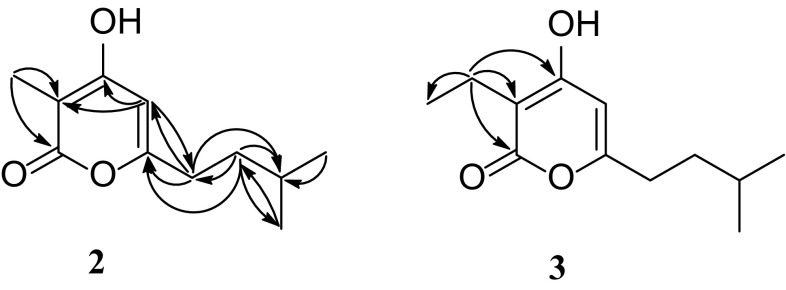
Key HMBC correlations of **2** and **3**

Violapyrone J (**2**) was obtained as a yellowish amorphous solid. And the molecular formula of **2** was determined to be C_11_H_16_O_3_ from the [M+H]^+^ peak at *m/z* 197.1176 (calcd for 197.1172) in the HRESIMS, indicating four degrees of unsaturation. Comparing with the ^1^H and ^13^C NMR data of violapyrone B (**1**) [19], a core structure of pyrone [19–21] of **1** with one olefinic proton (5.98, s) was evident. According to HSQC spectrum, two methylene carbons (*δ*_C_ 32.2 and 37.1), three methyl carbons (*δ*_C_ 8.3 and 22.3 × 2) and one *sp* ^3^ methine (*δ*_C_ 28.3) were determined. In the HMBC spectrum (Fig. 3), Me-11 (*δ*_H_ 1.83, s) correlated with C-2 (*δ*_C_ 169.3, CO) and C-3 (*δ*_C_ 98.8, C), indicating that the methyl was connected with C-3. The HMBC correlations from H-5 (*δ*_H_ 5.98, s) to C-3 (*δ*_C_ 98.8, C), C-4 (*δ*_C_ 168.4, C), C-7 (*δ*_C_ 32.2, CH_2_) and C-11 (*δ*_C_ 8.3, CH_3_), from H-7 (*δ*_H_ 2.47, t, *J* = 7.8 Hz) to C-5 (*δ*_C_ 101.1, CH), C-6 (*δ*_C_ 165.1, C), C-8 (*δ*_C_ 37.1, CH_2_) and C-9 (*δ*_C_ 28.3, CH), and from H-8 (*δ*_H_ 1.53, m) to C-6 (*δ*_C_ 165.1, C), C-7 (*δ*_C_ 32.2, CH_2_), C-9 (*δ*_C_ 28.3, CH) and C-10 (*δ*_C_ 22.3, CH_3_), suggested a connection of C-3/C-4/C-5/C-6/C-7/C-8/C-9. What’s more, in the HMBC spectrum, Me-10 (*δ*_H_ 0.95, d, *J* = 6.6 Hz) signal correlated with C-8 (*δ*_C_ 37.1, CH_2_) and C-9 (*δ*_C_ 28.3, CH), suggesting a carbon connection of C-8/C-9/C-10. In this way, the structure of **2** were confirmed (Fig. 1).

Violapyrone K (**3**) was also obtained as a yellowish amorphous solid. The HRESIMS data (*m/z* 211.1328 [M+H]^+^, calcd for 211.1329) of **3** showed the molecular formula C_12_H_18_O_3_, corresponding to four degrees of unsaturation. Analogously, a careful comparison of the NMR data between **2** and **3** (Table 1) reached a conclusion that an ethyl (*δ*_H_ 2.38, q, *J* = 7.2 Hz, 1.01, t, *J* = 7.2 Hz; *δ*_C_ 17.3, CH_2_, 13.1 CH_3_) appeared in **3** instead of a methyl (*δ*_H_ 1.83, s; *δ*_C_ 8.3, CH_3_) in **2**. This inference was verified by the HMBC correlations (Fig. 3) from H-11 (*δ*_H_ 2.38, q, *J* = 7.2 Hz) to C-2 (*δ*_C_ 169.3, C), C-3 (*δ*_C_ 104.9, C), C-4 (*δ*_C_ 168.4, C), and C-12 (*δ*_C_ 13.1, CH_3_), respectively. Therefore, the structure of **3** was established.

**Table 1:** ^1^H (600 MHz) and ^13^C NMR (150 MHz) data of **2** and **3** in CD_3_OD (*δ* in ppm)

No.	**2**		**3**	
	*δ*_C_, Type	*δ*_H_ (*J,* Hz)	*δ*_C_, Type	*δ*_H_ (*J*, Hz)
2	169.3, C		168.9, C	
3	98.8, C		104.9, C	
4	168.4, C		168.9, C	
5	101.1, CH	5.98, s	101.7, CH	5.95, s
6	165.1, C		165.2, C	
7	32.2, CH_2_	2.47, t (7.8)	32.3, CH_2_	2.46, t (7.8)
8	37.1, CH_2_	1.53, m	36.8, CH_2_	1.52, m
9	28.3, CH	1.58, m	28.8, CH	1.59, m
10	22.3, 2 × CH_3_	0.95, d (6.6)	22.6, 2 × CH_3_	0.94, d (6.6)
11	8.3, CH_3_	1.83, s	17.3, CH_2_	2.38, q (7.2)
12			13.1, CH_3_	1.01, t (7.2)

**Fig. 4:**
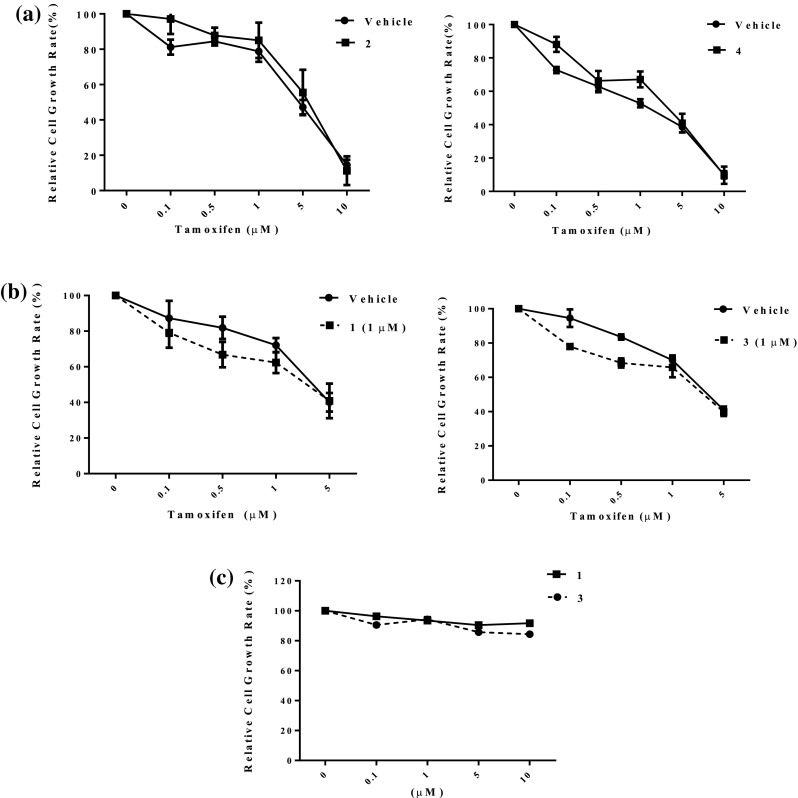
Effect of compounds **1**–**4** on cell growth and tamoxifen resistance in MCF-7/TamR. **a** Relative cell growth rate of MCF-7/TamR cells was tested with different doses of tamoxifen under the concentrations of 10, 15, and 20 μM of **2** (*left*) and **4** (*right*). **b** Relative cell growth rate of MCF-7/TamR cells was tested with different doses of tamoxifen under the minimum effect concentration of 1 μM of **1** (*left*) and **3** (*right*). **c** Relative cell growth rate of MCF-7/TamR was measured after 72 h treatment of indicated concentrations of violapyrones B (**1**) and K (**3**) (0.1–10 μM), respectively. Data was presented as mean ± SEM

The resensitization effect of four isolates **1–4** was further evaluated. The data suggested that violapyrones J (**2**) and I (**4**) showed no effect on cell growth upon tamoxifen treatment in MCF-7/TamR cells under the concentrations of 10, 15, and 20 μM (Fig. 4a). However, a combined treatment of either **1** or **3** with tamoxifen resulted in decreased cell growth rate in MCF-7/TamR cells at the concentration of 1 μM (Fig. 4b). In order to exclude the possibility of cytotoxic effect, we tested the cytotoxicity of violapyrones B (**1**) and K (**3**) in the absence of tamoxifen in multiple cancer cell lines. It was shown that both **1** and **3** exhibited no cytotoxicity against MCF-7/TamR (Fig. 4c), MCF-10A, MCF-7, SK-BR-3, MDA-MB-231, BGC-823 and A549 in our experiment (40 μM, 72 h) (Fig. S16).

Violapyrones B (**1**) and K (**3**) showed no cytotoxicity for MCF-7, MCF-7/TamR, MCF-10A, SK-BR-3, MDA-MB-231, BGC-823 and A549 cells at high concentration of 40 μM which is identical with the results that similar *α*-pyrone derivatives in the literature have no cytotoxicity [19], but it can improve the sensitivity of tamoxifen resistance for MCF-7/TamR at a low concentration of 1 μM. On the whole, our experimental data was persuasive. Prospectively, violapyrones B (**1**) and K (**3**) have possibilities to be further developed into a novel reversal agent in combination with tamoxifen for chemotherapy in breast cancer patients.

## Experimental Section

### General Experimental Procedures

NMR spectra were recorded in CD_3_OD using a Bruker AVANCE III-600 spectrometer, and TMS was used as internal standard. ESIMS spectra were recorded using a Waters Xevo TQS mass spectrometer. HRESIMS data were obtained using an Agilent 1290 UPLC/6540 Q-TOF mass instrument. Column chromatography (CC) was performed using silica gel (200–300 mesh, Qingdao Marine Chemical Inc., China) and MCI gel CHP20P (75–150 µm, Mitsubishi Corp., Japan). Semipreparative HPLC was conducted on a HITACHI Chromaster system equipped with a DAD detector, a YMC-Triart C_18_ column (250 × 10 mm i.d., 5 μm).

### Extraction and Isolation

The *Streptomyces* sp. KIB-H0495 was cultivated on a scale of 20 L using D fermentation medium (soluble starch 10 g, trypton 5 g, glucose 10 g, glycerol 10 g, yeast extract 5 g, CaCO_3_ 3 g in one liter distilled water, adjusted to pH 7.0 with 2 N NaOH) to cultivate at 30 °C for 7 days on a rotary shaker (200 rpm). The culture broth (total 20 L) was harvested by high speed centrifugation (6000 rpm) and then extracted with EtOAc (10 L ×3 times). The mycelium was extracted with acetone and the extract was concentrated in vacuo and the residual aqueous concentrate was then extracted with EtOAc. Both extracts revealed an identical set of metabolites based on HPLC analysis, therefore the extracts (5.1 g) were combined and then separated on silica gel [petroleum ether/EtOAc (1:0, 1:1, 1:3, 0:1) and CHCl_3_/CH_3_OH (1:1)] CC, yielding five fractions A-E. Fraction B (1.93 g) was separated on MCI CC eluting with gradient CH_3_OH/H_2_O (35, 50, 75, 85, 100% CH_3_OH). From 50% CH_3_OH/H_2_O part, compounds **1** (2.2 mg), **2** (1.0 mg), **3** (1.1 mg), and **4** (1.3 mg) were obtained after a preparation by semipreparative HPLC eluting with 68% CH_3_OH/H_2_O at the flow rate of 3.0 mL/min.

Violapyrone J (**2**): Yellowish amorphous solid; UV (MeOH) *λ*_max_ (log *ε*) 204 (4.3), 289 (3.9) nm; ^1^H and ^13^C NMR see Table 1; ESIMS *m/z* 197 [M+H]^+^; HRESIMS 197.1176 (C_11_H_17_O_3_, calcd for 197.1172).

Violapyrone K (**3**): Yellowish amorphous solid; UV (MeOH) *λ*_max_ (log *ε*) 204 (4.2), 289 (3.9) nm; ^1^H and ^13^C NMR see Table 1; ESIMS *m/z* 211 [M+H]^+^; HRESIMS 211.1328 (C_12_H_19_O_3_, calcd for 211.1329).

### Cell Culture

Parental breast cancer cell line MCF-7 and its derived tamoxifen resistant lines MCF-7/TamR were kfindly provided by Dr. Tao Zhu (University of science and technology of China, China) and cultured as described before [22]. Breast cancer cells SK-BR-3 and gastric cancer cells BGC-823 were cultured in RPMI 1640 (Invitrogen/Life Technologies) medium with 2.0 g/L NaHCO_3_ (Sigma, USA); A549 was cultured in DMEM medium; Breast cancer cells MDA-MB-231 were cultured in DMEM/F12 medium with 1.2 g/L NaHCO_3_. All the above mentioned medium were also supplemented with 10% Fetal bovine serum (FBS) (Gibco, Invitrogen, USA) and 1% penicillin–streptomycin (WISENT, China). Normal breast epithelial cells MCF-10A were cultured in DMEM/F12 medium with 1.2 g/L NaHCO_3_, 5% horse serum (Gibco, USA), 10 μg/mL insulin (Roche, Switzerland), 20 ng/mL EGF2 (Sigma, USA), 100 ng/mL cholera toxin α subunit (Sigma, USA) and 0.5 μg/mL hydrocortisone (Melonepharma, China).

### Cytotoxicity Assay

Cytotoxicity of KIB-H0495 extract was investigated using resistant cell line MCF-7/TamR. Four *α*-pyrone derivatives were tested alone against breast epithelial cells (MCF-10A, MCF-7), tamoxifen resistant cells (MCF-7/TamR), breast cancer cells (SK-BR-3, MDA-MB-231), gastric cancer cells (BGC-823) and lung cancer cells (A549). The cytotoxicity of MCF-7/TamR was detected by inputting different concentrations of tamoxifen under separately quantitative compounds **1**–**4** and DMSO.

### Sulforhodamine B (SRB) Assay

The SRB assay was modified according to the method described by Pauwels et al. [23]. Briefly, 3000 cells/well were seeded in 96-well plates for 24 h before receiving vehicle or tamoxifen treatment. 72 h later, the culture medium was aspirated, and the remaining cells were fixed with 10% Trichloroacetic acid (TCA) at 4  °C (200 μL/well) for 1 h. Cells were then washed five times with 100 μL deionized water, and left to air-dry at room temperature. The cells were next stained with 100 μL 0.4% SRB (Sigma, USA) at 37 °C for 30 min, and subsequently washed five times with 1% acetic acid to remove unbound stain. Lastly, the plates were dried at 37 °C and bound protein stain was solubilized with 100 μL of 10 mM unbuffered Tris base (Sigma, USA) on shaker for 30 min. Afterwards, the 96-well plates were transferred to CLARIO star microplate reader (BMG LABTECH, Germany) to detect optical density (OD) at 515 nm. The formulae below was used to calculate the relative cell growth rate [24].

$${\text{Relative cell growth rate }}\left( \% \right) = {{\left( {{\text{mean OD}}_{\text{sample}} {-}{\text{mean OD}}_{\text{day1}} } \right)} \mathord{\left/ {\vphantom {{\left( {{\text{mean OD}}_{\text{sample}} {-}{\text{mean OD}}_{\text{day1}} } \right)} {\left( {{\text{mean OD}}_{\text{negative control}} {-}{\text{mean OD}}_{\text{day1}} } \right)}}} \right. \kern-0pt} {\left( {{\text{mean OD}}_{\text{negative control}} {-}{\text{mean OD}}_{\text{day1}} } \right)}}\%$$

## Electronic Supplementary Material

Below is the link to the electronic supplementary material.

Supplementary material 1 (PDF 658 kb)
