# Supplementary material for: α-Pyrone Derivatives from a Streptomyces Strain Resensitize Tamoxifen Resistance in Breast Cancer Cells
Source: Nat Prod Bioprospect. 2017 Jun 20;7(4):329–34. doi: 10.1007/s13659-017-0136-8 (PMC5507810; doi:10.1007/s13659-017-0136-8)
Supplement: Supplementary file 1 — Supplementary material 1 (PDF 658 kb) [file 13659_2017_136_MOESM1_ESM.pdf]

# Supplementary Material

## *α*-Pyrone Derivatives from a *Streptomyces* Strain Resensitize Tamoxifen Resistance in Breast Cancer Cells

Ruimin Yang, Xiulei Zhang, Li Wang, Jianping Huang, Jing Yang, Yijun Yan, Jianying Luo

Xiangting Wang*, and Sheng-Xiong Huang*

$^{1}$H and $^{13}$C NMR and ESIMS data of compounds **1** and **4**

**Fig. S1** $^{1}$H NMR spectrum of violapyrone B (**1**) in $CD_3OD$ (600 MHz).

**Fig. S2** $^{13}$C NMR spectrum of violapyrone B (**1**) in $CD_3OD$ (150 MHz)

**Fig. S3** $^{1}$H NMR spectrum of violapyrone J (**2**) in $CD_3OD$ (600 MHz).

**Fig. S4** $^{13}$C NMR spectrum of violapyrone J (**2**) in $CD_3OD$ (150 MHz).

**Fig. S5** HSQC NMR spectrum of violapyrone J (**2**) in $CD_3OD$

**Fig. S6** HMBC NMR spectrum of violapyrone J (**2**) in $CD_3OD$

**Fig. S7** HRESIMS spectrum of violapyrone J (**2**)

**Fig. S8** $^{1}$H NMR spectrum of violapyrone K (**3**) in $CD_3OD$ (600 MHz).

**Fig. S9** $^{13}$C NMR spectrum of violapyrone K (**3**) in $CD_3OD$ (150 MHz)

**Fig. S10** HSQC NMR spectrum of violapyrone K (**3**) in $CD_3OD$

**Fig. S11** HMBC NMR spectrum of violapyrone K (**3**) in $CD_3OD$

**Fig. S12** HRESIMS spectrum of violapyrone K (**3**)

**Fig S13** $^{1}$H NMR spectrum of violapyrone I (**4**) in $CD_3OD$ (600 MHz).

**Fig. S14** $^{13}$C NMR spectrum of violapyrone I (**4**) in $CD_3OD$ (150 MHz)

**Fig. S15** Relative cell growth rate of MCF-7 and MCF-7/TamR cells treated with different concentrations of tamoxifen

**Fig. S16** Cytotoxic activities of violapyrones B (**1**) and K (**3**) on different cancer cell lines

**$^1$H and $^{13}$C NMR and ESIMS data of compounds 1 and 4**

Violapyrone B (**1**): Yellowish amorphous solid. $^1$H NMR (600 MHz, $CD_3OD$): $\delta_H$ 5.96 (1H, s, H-5), 2.45 (2H, t, *J* = 7.8 Hz, H-7), 1.84 (3H, s, Me-3), 1.61 (2H, m, H-8), 1.53 (1H, m, H-11), 1.35 (2H, m, H-9), 1.21 (2H, m, H-10), 0.88 (6H, d, *J* = 7.2 Hz, H-12); $^{13}$C NMR ($CD_3OD$): $\delta_C$ 169.3 (C-2), 168.9 (C-4), 164.7 (C-6), 101.5 (C-5), 98.9 (C-3), 39.7 (C-10), 34.2 (C-7), 29.0 (C-11), 28.1 (C-8), 27.7 (C-9), 22.9 (C-12), 8.2 (Me-3).

Violapyrone I (**4**) : $^1$H NMR (600 MHz, $CD_3OD$): $\delta_H$ 5.93 (1H, s, H-5), 2.44 (2H, t, *J* = 7.8 Hz, H-7), 1.83 (3H, s, 3-Me), 1.63 (2H, m, H-8), 1.34 (2H, m, H-10), 1.33 (2H, m, H-9), 1.31 (2H, m, H-12), 1.30 (2H, m, H-11), 0.89 (3H, t, *J* = 6.6 Hz, H-13); $^{13}$C NMR (150 MHz, $CD_3OD$): $\delta_C$ 170.4 (C-2), 169.5 (C-4), 164.5 (C-6), 102.3 (C-5), 98.3 (C-3), 34.2 (C-7), 32.8 (C-11), 30.0 (C-9), 29.9 (C-10), 27.9 (C-8), 23.6 (C-12), 14.3 (C-13), 8.3 (3-$CH_3$); positive ESIMS: *m/z* 247 $[M + Na]^+$.

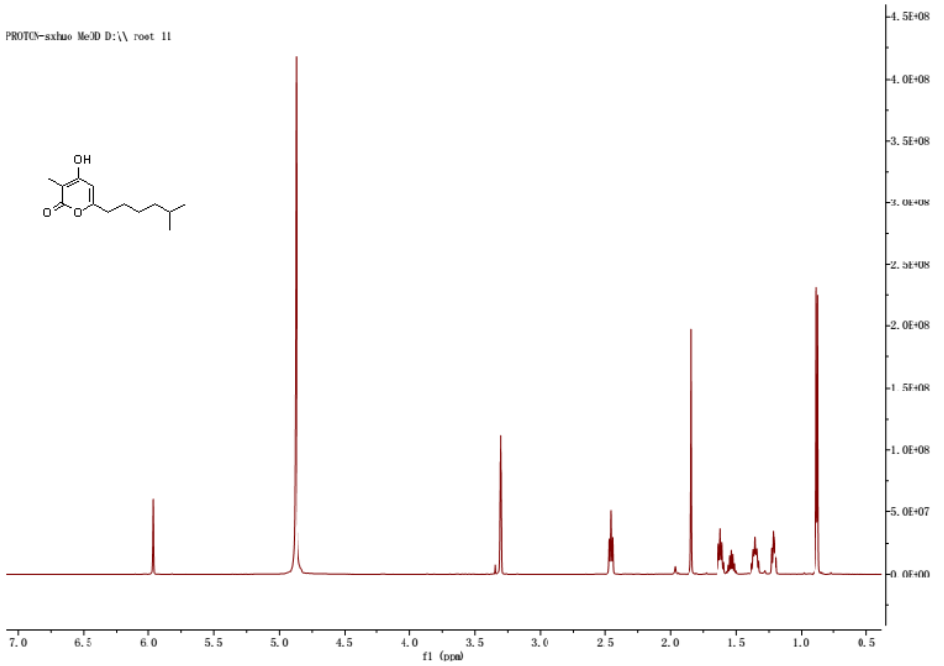


**Fig. S1** $^{1}$H NMR spectrum of violapyrone B (**1**) in $CD_3OD$ (600 MHz).

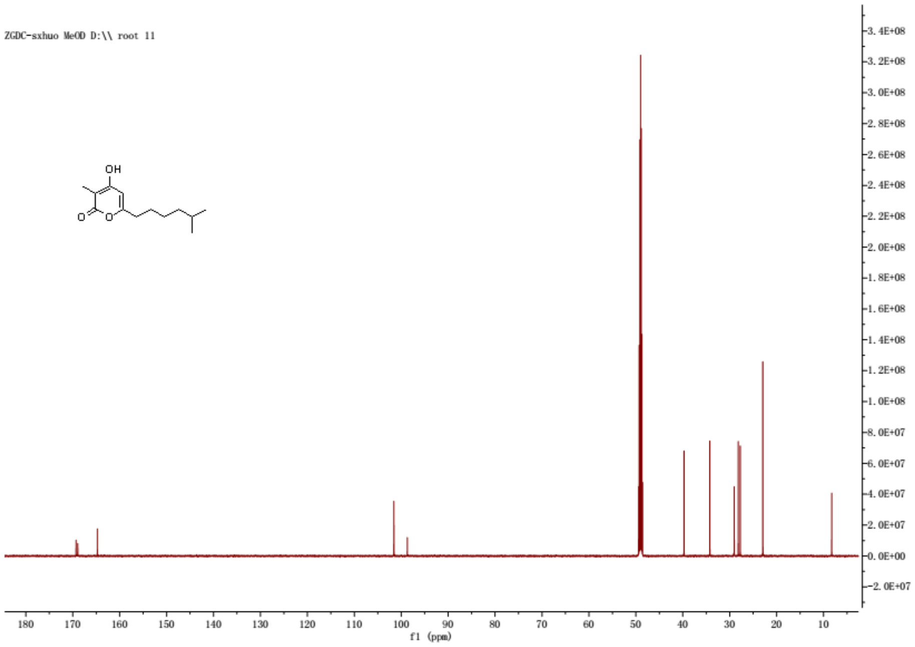


**Fig. S2** $^{13}$C NMR spectrum of violapyrone B (**1**) in $CD_3OD$ (150 MHz).

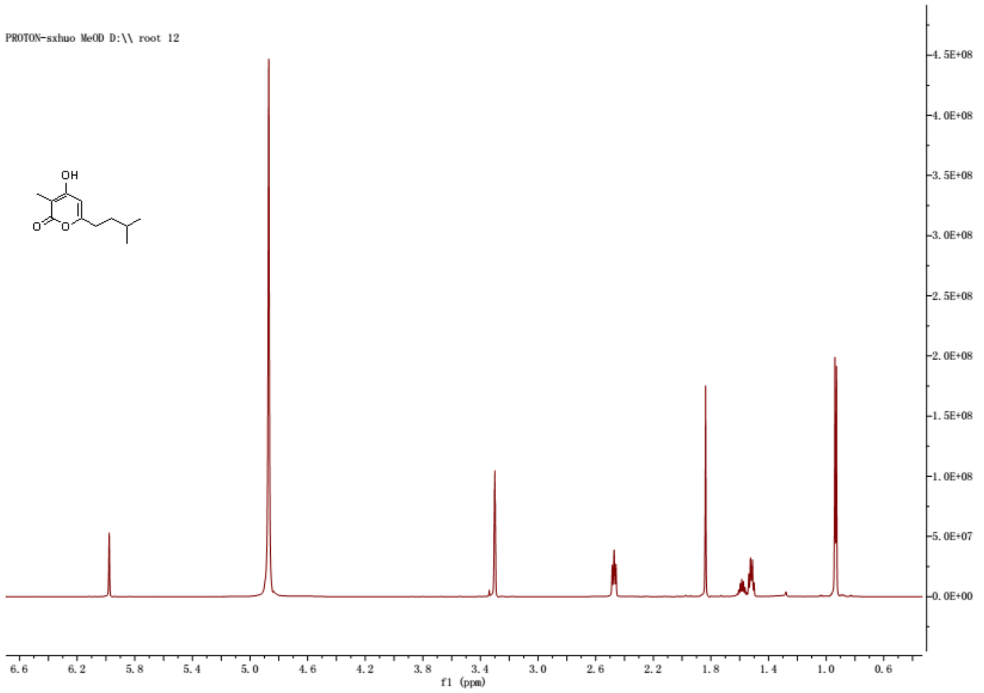


**Fig. S3** $^{1}$H NMR spectrum of violapyrone J (**2**) in $CD_3OD$ (600 MHz).

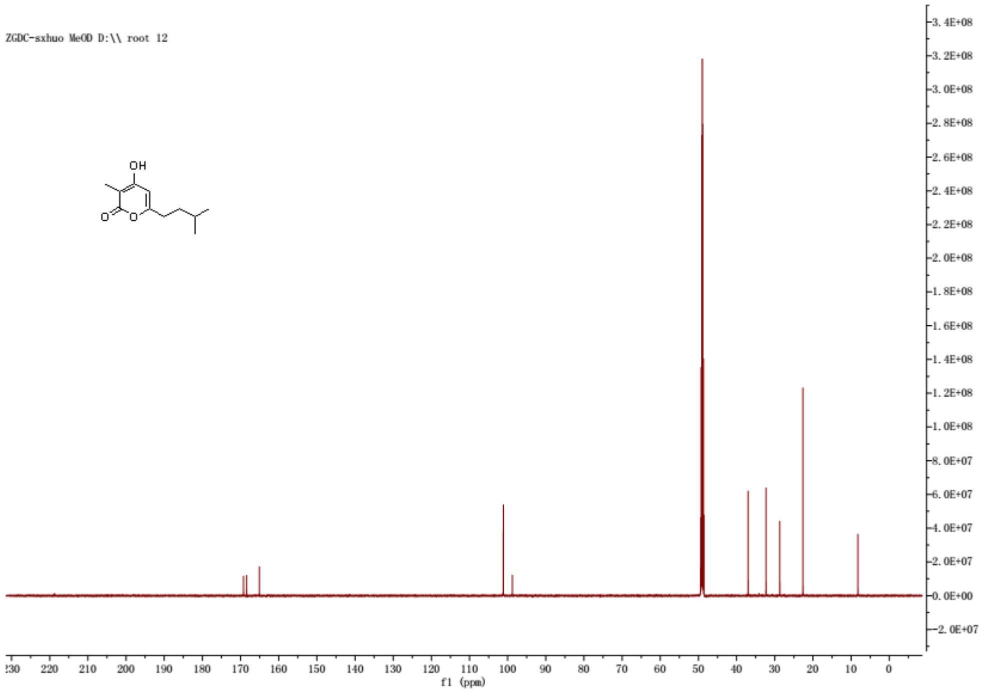


**Fig. S4** $^{13}$C NMR spectrum of violapyrone J (**2**) in $CD_3OD$ (150 MHz).

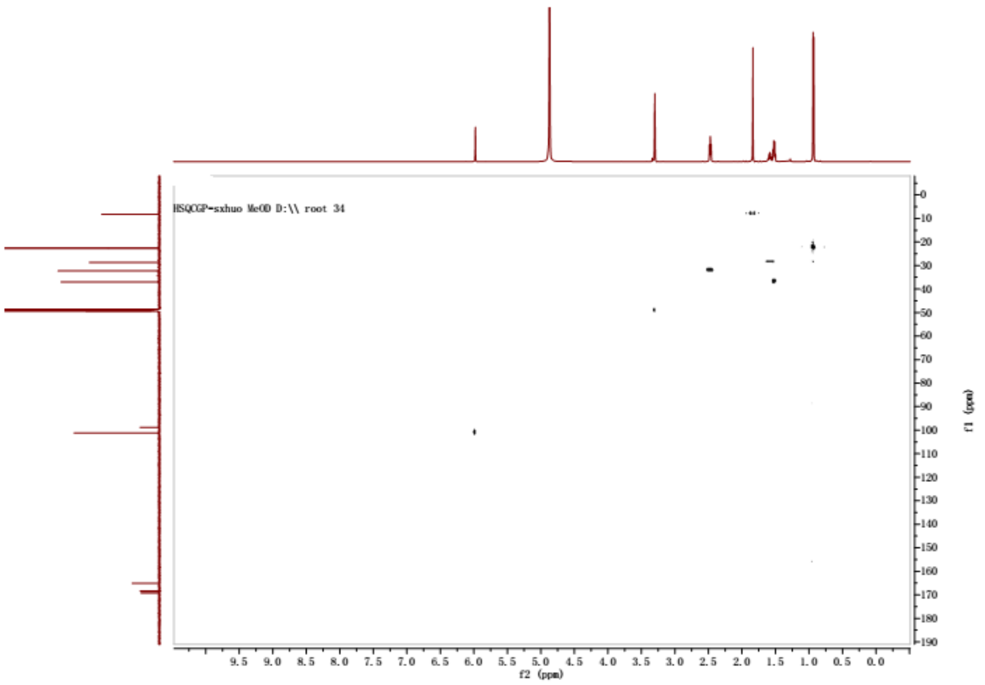


**Fig. S5** HSQC NMR spectrum of violapyrone J (**2**) in $CD_3OD$

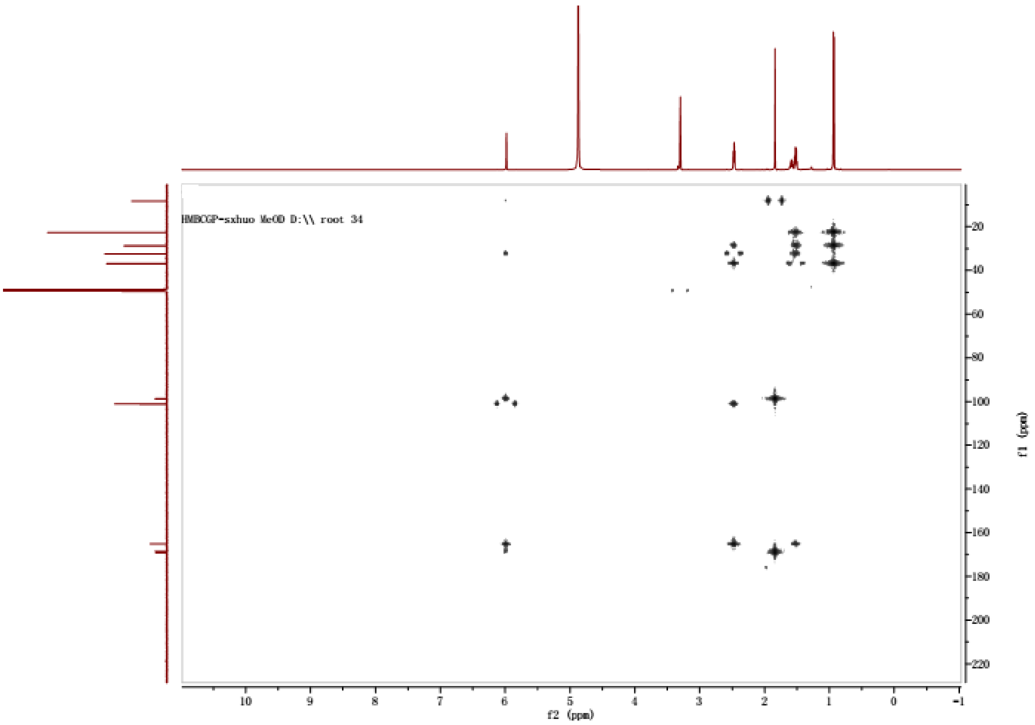


**Fig. S6** HMBC NMR spectrum of violapyrone J (**2**) in $CD_3OD$

**User Spectra**

| Fragmentor Voltage | Collision Energy | Ionization Mode |
|---|---|---|
| 135 | 0 | ESI |

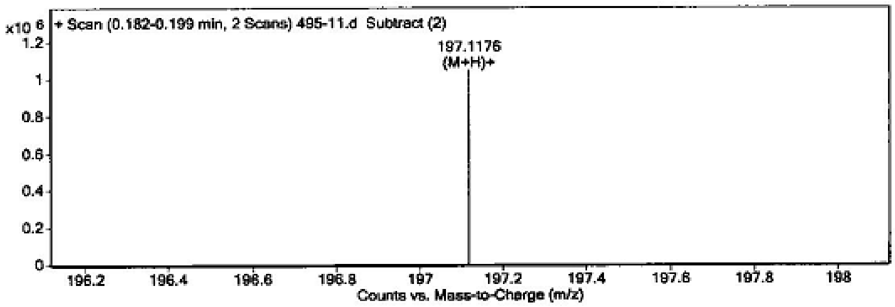


**Peak List**

| *m/z* | z | Abund | Formula | Ion |
|---|---|---|---|---|
| 197.1176 | 1 | 1050815.25 | C11 H16 O3 | (M+H)+ |
| 198.1207 | 1 | 110631.98 | C11 H16 O3 | (M+H)+ |
| 219.0993 | 1 | 258063.84 | | |
| 235.0733 | 1 | 46053.26 | | |
| 244.0969 | 1 | 67566.75 | | |
| 276.1231 | 1 | 134696.45 | | |
| 298.1053 | 1 | 101462.34 | | |
| 415.2092 | 1 | 49013.46 | | |

**Formula Calculator Element Limits**

| Element | Min | Max |
|---|---|---|
| C | 3 | 60 |
| H | 0 | 120 |
| O | 0 | 30 |
| N | 0 | 30 |

**Formula Calculator Results**

| Formula | CalculatedMass | CalculatedMz | Mz | Diff. (mDa) | Diff. (ppm) | DBE |
|---|---|---|---|---|---|---|
| C11 H16 O3 | 196.1099 | 197.1172 | 197.1176 | -0.3 | -1.7 | 4.0000 |

**Fig. S7** HRESIMS spectrum of violapyrone J (**2**)

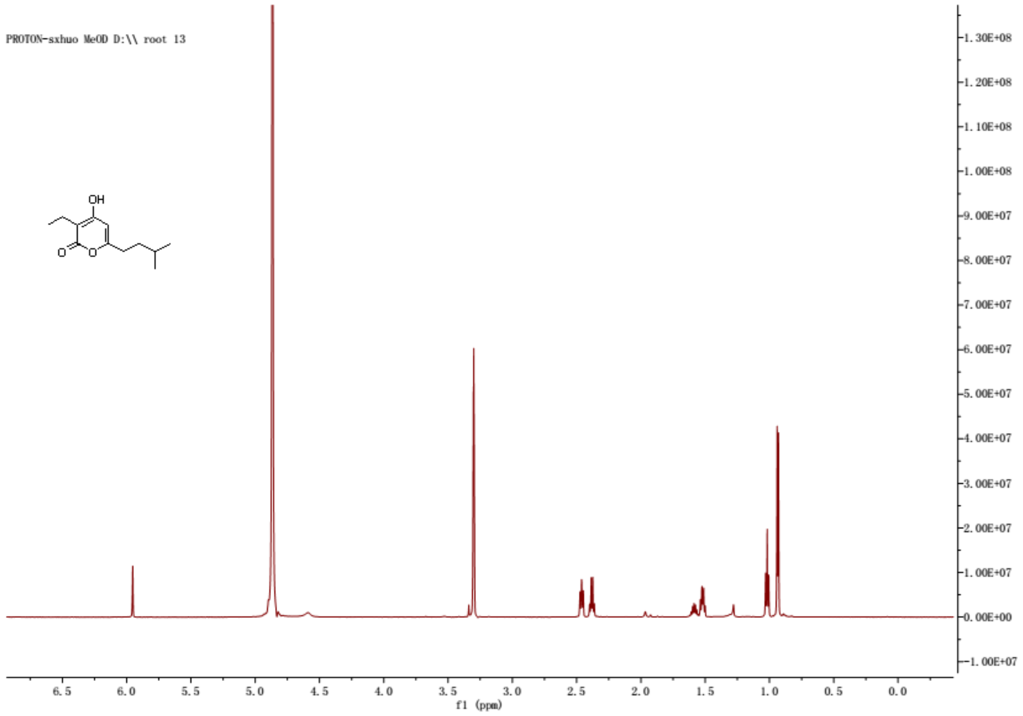


**Fig. S8** $^{1}$H NMR spectrum of violapyrone K (**3**) in $CD_3OD$ (600 MHz).

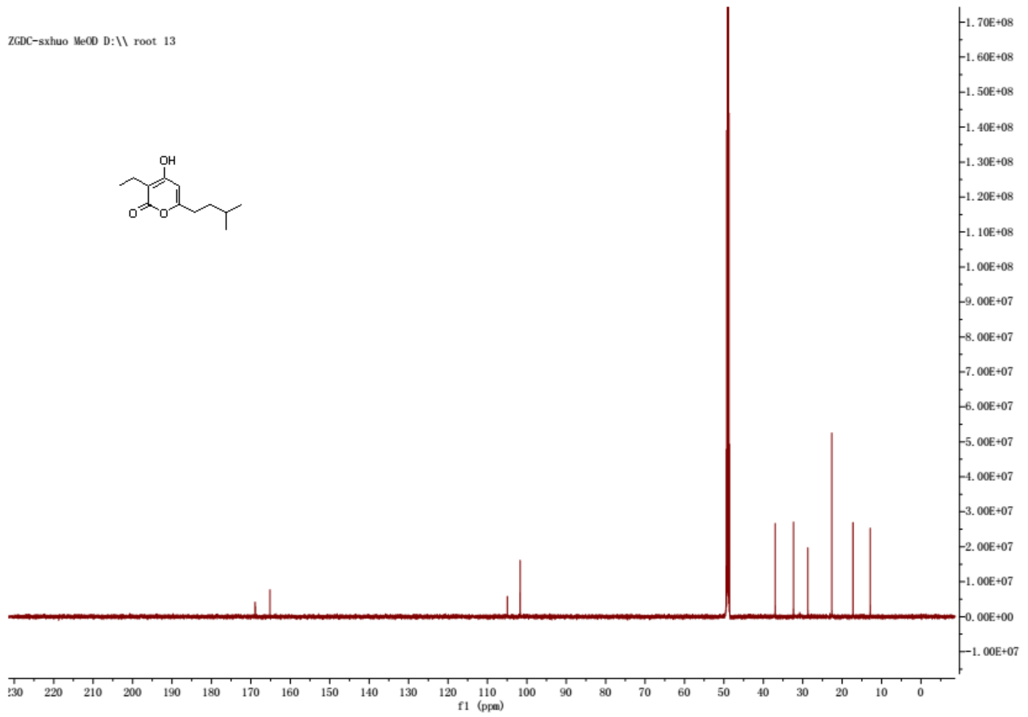


**Fig. S9** $^{13}C$ NMR spectrum of violapyrone K (**3**) in $CD_3OD$ (150 MHz)

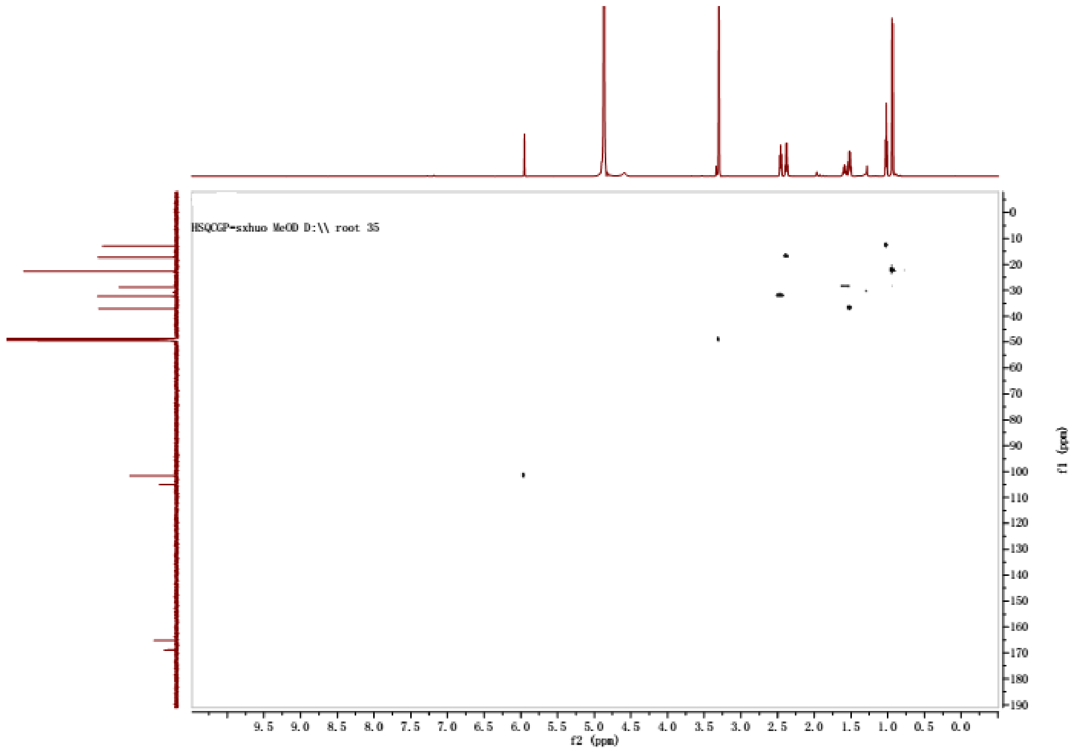


**Fig. S10** HSQC NMR spectrum of violapyrone K (**3**) in $CD_3OD$

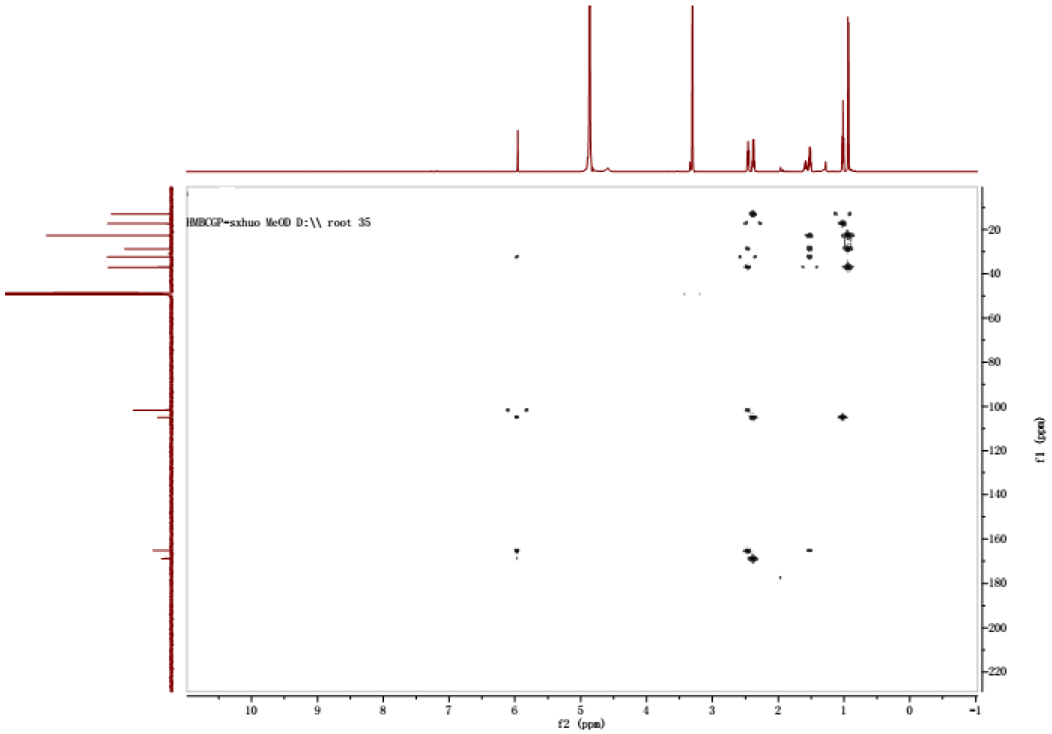


**Fig. S11** HMBC NMR spectrum of violapyrone K (**3**) in $CD_3OD$

**User Spectra**

| Fragmentor Voltage | Collision Energy | Ionization Mode |
|---|---|---|
| 135 | 0 | ESI |

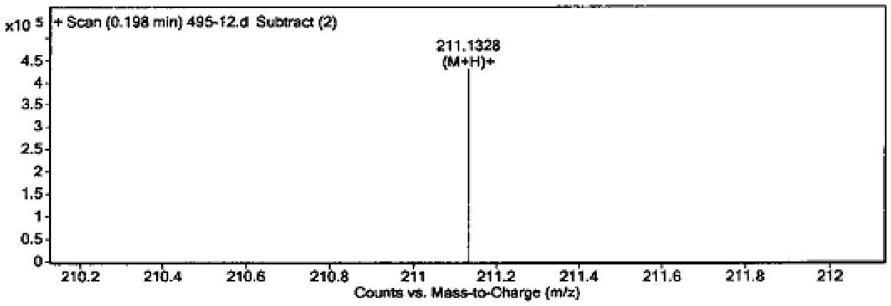


**Peak List**

| *m/z* | z | Abund | Formula | Ion |
|---|---|---|---|---|
| 166.0709 | 2 | 8198.85 | | |
| 211.1328 | 1 | 432262.47 | C12 H18 O3 | (M+H)+ |
| 212.136 | 1 | 57055.99 | C12 H18 O3 | (M+H)+ |
| 233.1146 | 1 | 122387.84 | | |
| 234.1177 | 1 | 16257.97 | | |
| 249.089 | 1 | 24724.87 | | |
| 421.2579 | 1 | 9978.59 | | |
| 443.2407 | 1 | 12264.39 | | |

**Formula Calculator Element Limits**

| Element | Min | Max |
|---|---|---|
| C | 3 | 60 |
| H | 0 | 120 |
| O | 0 | 30 |
| N | 0 | 30 |

**Formula Calculator Results**

| Formula | CalculatedMass | CalculatedMz | Mz | Diff. (mDa) | Diff. (ppm) | DBE |
|---|---|---|---|---|---|---|
| C12 H18 O3 | 210.1256 | 211.1329 | 211.1328 | 0.1 | 0.3 | 4.0000 |

**Fig. S12** HRESIMS spectrum of violapyrone K (**3**)

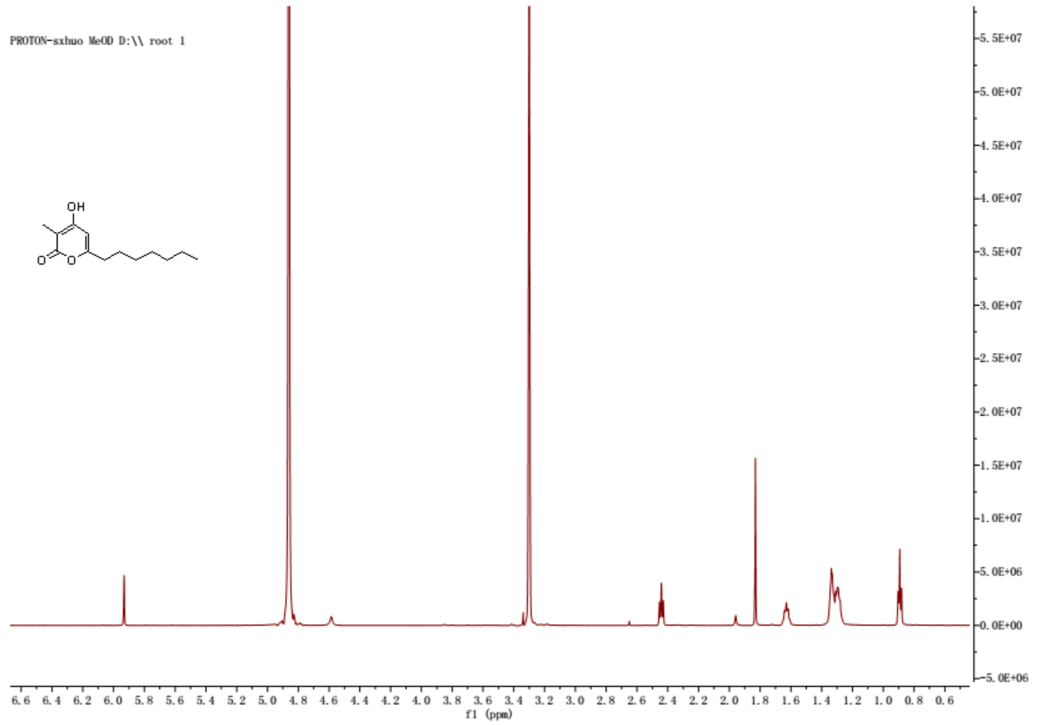


**Fig. S13** $^{1}$H NMR spectrum of I (**4**) in $CD_3OD$ (600 MHz).

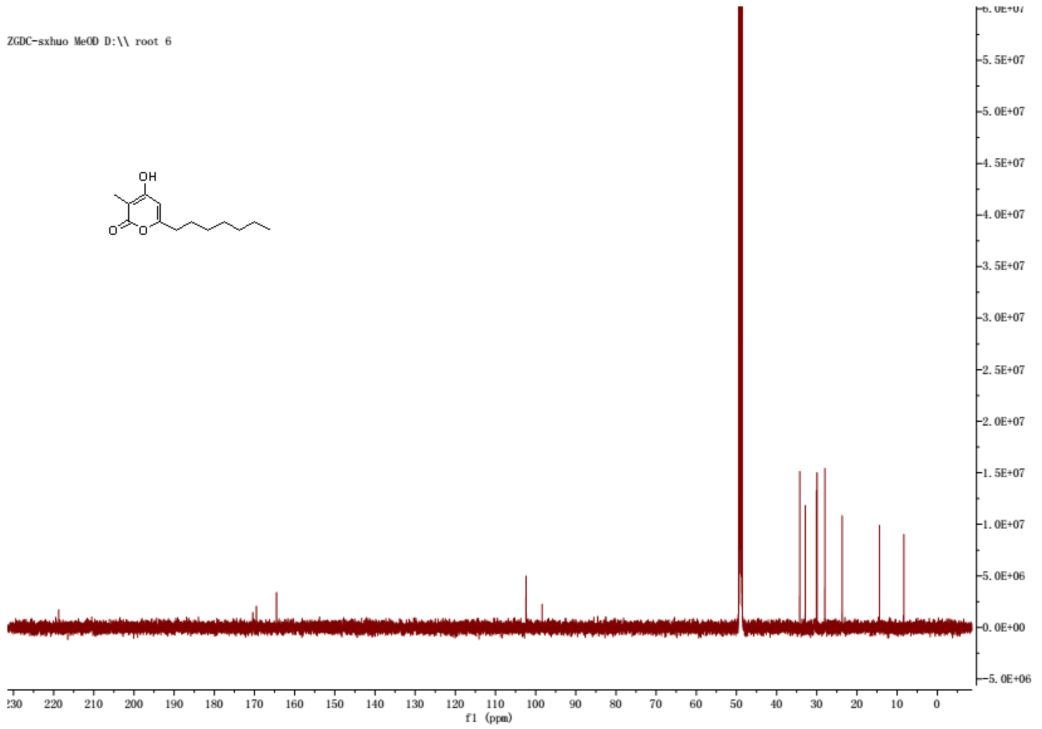


**Fig. S14** $^{13}$C NMR spectrum of I (**4**) in $CD_3OD$ (150 MHz)

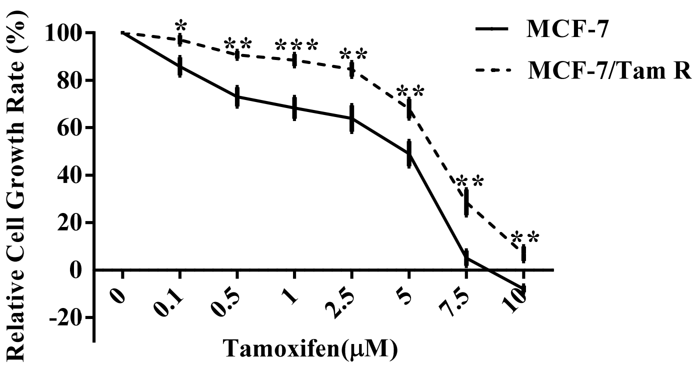


**Fig. S15** Relative cell growth rate of MCF-7 and MCF-7/TamR cells treated with different concentrations of tamoxifen (0.1–10 μM) in 72 h. Data was presented as means ± SEM, *P < 0.05, **P < 0.01, ***P < 0.001

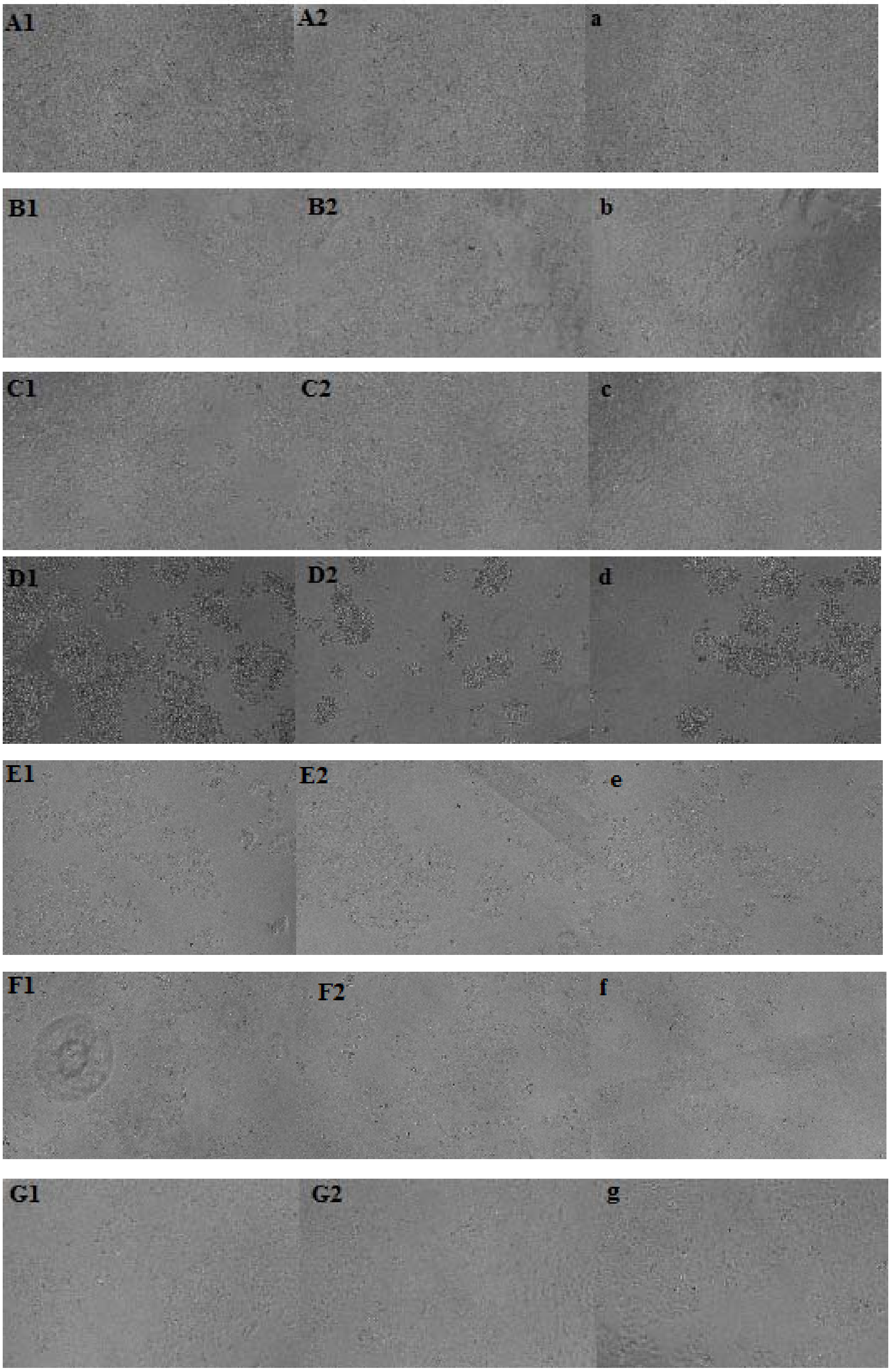


**Fig. S16** Cytotoxic activities of violapyrones B (**1**) and K (**3**) on MCF-10A (A1, A2), MCF-7 (B1, B2), MCF-7/Tam R (C1, C2), SK-BR-3 (D1, D2), MDA-MB-231 (E1, E2), BGC-823 (F1, F2) and A549 (G1, G2) after 72 h treatment (40 μM) compared to their respective control group (a, b, c, d, e, f, g)
